# Is the 32-kDa fragment the functional enamelin unit in all species?

**DOI:** 10.1111/j.1600-0722.2011.00869.x

**Published:** 2011-12

**Authors:** Steven J Brookes, Nicola J Kingswell, Martin J Barron, Michael J Dixon, Jennifer Kirkham

**Affiliations:** 1 Department of Oral Biology, Leeds Dental Institute, University of LeedsLeeds, UK; 2 Faculty of Life Sciences and School of Dentistry, University of ManchesterManchester, UK

**Keywords:** 32-kDa enamelin, amelogenesis, biomineralization, enamel matrix

## Abstract

Enamelin is an extracellular enamel matrix protein essential for normal amelogenesis. After secretion, porcine enamelin is processed to generate several enamelin-degradation products. The cumulative 32-kDa enamelin is the most abundant enamelin present, and various roles for this molecule have been suggested. However, the proteolytic cleavage sites in porcine enamelin that generate the 32-kDa enamelin are not conserved across species, and the 32-kDa enamelin analogue may not be present in all species. To explore this we studied rat enamelin biochemistry using western blotting with anti-peptide IgGs to porcine 32-kDa enamelin and to the putative rat 32-kDa enamelin analogue. The dominant enamelins in secretory-stage rat enamel migrated at around 60–70 kDa. In contrast, the dominant enamelins in secretory-stage porcine enamel migrated at around 32 kDa. In contrast, secretory-stage porcine-enamel enamelins were dominated by the 32-kDa enamelin. Rat enamelin was completely removed from maturation-stage enamel without any accumulation of 32-kDa enamelin. We suggest that a discrete 32-kDa enamelin is not essential for normal amelogenesis in all species, and in pig it may be a processing product of a larger functional enamelin molecule. The pig may be an atypical model in terms of enamelin biochemistry and function, and caution should be exercised when assigning functional roles to the 32-kDa enamelin as a discrete enamel matrix entity.

Dental enamel is the hardest substance found in biology. It is composed of greatly elongated hydroxyapatite mineral crystals whose ordered architectural deposition within the tissue is exquisitely controlled during enamel formation by a protein extracellular matrix secreted by the enamel-forming ameloblasts. More than 90% of this matrix is derived from the amelogenin gene, with the bulk of the remainder being derived from the ameloblastin and enamelin genes. Trace amounts of proteolytic enzymes are also present. During the secretory stage of enamel formation, when the enamel layer is being deposited and is partially mineralized, all of the newly secreted enamel matrix proteins are subjected to extracellular proteolytic processing, which occurs through the action of enamelysin [matrix metalloproteinase 20 (MMP20)] (1, 2). Once the enamel has reached its final thickness, the protein matrix is removed proteolytically under the action of kallikrein-related peptidase 4 (1, 2) and the individual hydroxyapatite crystals elaborated during secretion grow in width and thickness so that the tissue is occluded with mineral. Although a minor component of the enamel matrix, enamelin appears to be essential for correct enamel formation. Enamelin mutations in humans cause autosomal-dominant amelogenesis imperfecta (3–7). Animal studies based on *N*-ethyl-*N*-nitrosourea induced dominant mouse mutations support observations in humans (8), and enamelin knockout mice (9) show that an absence of enamelin results in a severely affected enamel phenotype with only a very thin layer of mineralized tissue covering the erupted dentine.

Enamelin biochemistry has been mostly studied in the pig because of the ready availability of developing porcine teeth (from the meat industry) and the relatively large amount of developing enamel matrix that can be obtained from these teeth. Based on the primary porcine enamelin sequence, the predicted molecular mass of the newly secreted enamelin molecule is around 124 kDa, whereas its apparent molecular mass following SDS-PAGE is about 186 kDa (10). This apparent discrepancy is caused by post-translational glycosylation, with the majority of the glycosylation being concentrated in a region of the porcine enamelin molecule that generates the so-called ‘32-kDa enamelin’(11). This enamelin fragment, corresponding to Leu174 to Arg279 of the newly translated porcine enamelin, is generated by proteolytic matrix processing (12).

Like all enamel matrix proteins, porcine enamelin experiences a series of proteolytic cleavages as soon as it is secreted, so that the full-length, nascent molecule is only found in the newly secreted surface layer of enamel subadjacent to the ameloblasts. In the deeper, older enamel layers, the proportion of enamelin processing products increases, although it is the 32-kDa enamelin that is the most abundant enamelin-derived protein in the porcine developing enamel matrix, accounting for about 1% of the total protein (12–14).

Given its relative abundance in porcine enamel matrix, it is no surprise that most of the work carried out on enamelin has focused on the 32-kDa enamelin processing product (12). For example, the hydroxyapatite-binding characteristics of the 32-kDa enamelin, and its ability to inhibit hydroxyapatite crystal growth, have been investigated (14). It has been suggested that the mineral-bound 32-kDa enamelin may control enamel crystallite growth in the deeper enamel layer (15). An elegant series of studies has elucidated the post-translational glycosylation of the 32-kDa enamelin (11), and the influence of this glycosylation on its proteolytic degradation by MMP-20 and kallikrein-related peptidase 4 has been investigated (16). It has been proposed that the 32-kDa enamelin and amelogenins co-operate to promote enamel mineral nucleation and that among other enamel proteins of the extracellular matrix, the 32-kDa enamelin is the most appropriate candidate to act as the crystal nucleator of enamel apatite (17).

However, sequence comparisons between porcine enamelin and enamelin from several other species, including rat, mouse, and human, show that the cleavage sites responsible for generating the porcine 32-kDa enamelin are not fully conserved (Fig. 1). In rat, mouse, and human, the domain is present but the peptide bonds that would have to be hydrolyzed to generate the analogous 32-kDa enamelin in these species would lie between Pro and Pro to generate the N-terminus (rather than between Pro and Leu, as in pig) and between Gly and Arg (rather than between Arg and Ser, as in pig) to generate the C-terminus (Fig. 1). It is possible that the proteolytic enzyme activity responsible for generating the porcine 32-kDa enamelin does not exhibit a high degree of specificity and is also active against rat, mouse, and human enamelin and therefore still able to generate a processing product analogous to the porcine 32-kDa enamelin in these non-porcine species.

**Fig. 1:**
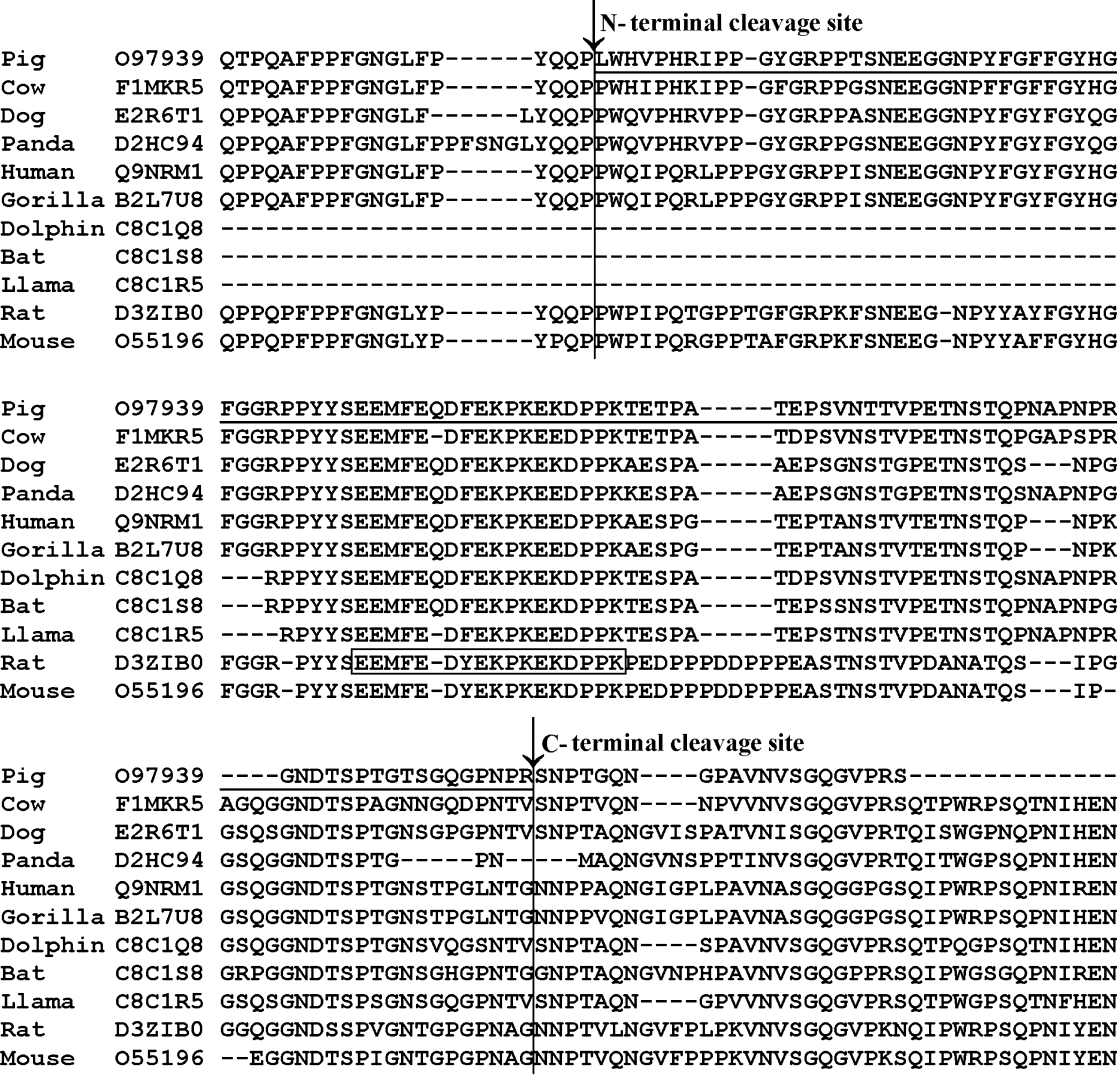
Alignment of the porcine enamelin domain giving rise to the 32-kDa enamelin molecule. The N- and C-terminal cleavage sites are not conserved across species. The boxed sequence shows the location of the peptide used to raise the anti-32 kDa IgGs.

In order to address this issue, we formulated the null hypothesis that the generation of the porcine 32-kDa enamelin fragment is an important step in enamel formation and as such plays a role in enamel development such that an analogous molecule of similar size and sequence will be generated in all species, even if the cleavage sites responsible for its generation are not conserved in these other species. To test this hypothesis, antibodies were raised to a synthetic peptide corresponding to a sequence located in the central region of what we assume would be the rodent analogue of the porcine 32-kDa enamelin (Fig. 2). Developing rat enamel was then probed using these antibodies to characterize the rat extracellular enamel matrix in terms of its enamelin composition. This is an important point because it raises the possibility that porcine developing enamel may not be representative of other species in terms of extracellular matrix enamelin biochemistry.

**Fig. 2:**
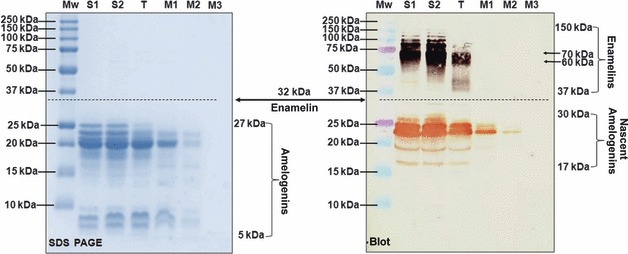
SDS-PAGE and western blotting of rat enamel matrix proteins from early secretory stage (S1), late secretory stage (S2), transition stage (T), early maturation stage (M1), midmaturation stage (M2), and late maturation stage (M3). The SDS-polyacrylamide gel shows the obvious removal of all protein during the maturation stage. The western blot is dual stained for both enamelin (brown/black staining) and nascent amelogenin (red staining). The brown/black staining shows that the dominant enamelin species present is migrating at 60–70 kDa, with no prominent staining visible at the predicted molecular mass of the 32-kDa enamelin (as indicated by the dotted line). During the transition stage (T), further enamelin processing appears to generate a smear of degradation products migrating between the 37- and 50-kDa molecular mass markers. Enamelin is absent from the maturation-stage enamel (M1–M3). Nascent amelogenin expression, as indicated by the red staining, shows that amelogenin and enamelin expression, extracellular processing, and removal from the tissue occur in the same temporal time frame. Mw, molecular-weight-marker ladder.

## Material and methods

### Source of enamel

Developing rat enamel was obtained from the lower incisors of 150 g male Wistar rats. The rats were killed by CO_2_ inhalation, and the lower incisor teeth were dissected free of the lower mandibles. The enamel organ was gently removed with a moist paper tissue and the tooth was air-dried for 1 min until the white opaque zone, representing the start of the maturation stage of amelogenesis, became visible (18). Secretory-stage and transition-stage enamel samples, measuring 1.5–2 mm, were dissected apical to the white opaque zone. The white opaque zone and remaining maturation-stage enamel were similarly dissected. The resulting samples – early secretion (S1), late secretion (S2), transition (T), early maturation (M1), midmaturation (M2), and late maturation (M3) – were stored at −80°C until required.

Developing porcine tooth germs were obtained from animals approximately 6 months of age. The pigs were killed by anaesthetic overdose and the molar teeth were removed from the lower mandibles. The enamel organ and pulp tissues were removed and the enamel was wiped with a moist paper tissue. The soft secretory-stage enamel was scraped off the dentine and stored at −80°C until required.

### Identification of enamelin processing products generated during rat enamel development

The series of enamel samples representing all stages of rat incisor enamel development (S1, S2, T, M1, M2, and M3) were extracted by grinding in 15 μl of 100 mM phosphate buffer (pH 7.4) with a fine glass rod. The extracts were clarified by centrifugation and the supernatants were removed. The pellet was then extracted with 15 μl of double-concentrate SDS-PAGE sample loading buffer (pH 6.8). The SDS extracts were clarified by centrifugation and added to the phosphate-buffer extracts. This double-extraction technique effectively solubilizes all enamel matrix proteins and is equivalent to protein extraction based on acid demineralization of the tissue (15). As a comparison, secretory-stage porcine enamel was similarly extracted using 50 μl of each extraction solution per mg of enamel.

All samples were heated at 90°C for 2 min, loaded at 10 μl per lane, and resolved at 200 V on 1-mm-thick 12% SDS-PAGE minigels (Mini Protean III; Bio-Rad, Hemel Hempstead, UK). Then, the gels were fixed and stained with Coomassie Blue G250. Duplicate gels were blotted onto nitrocellulose (Mini Trans-Blot; Bio-Rad) at 60 V for 1 h. The blots were blocked overnight in Tris-buffered saline (TBS; 20 mM Tris and 500 mM NaCl, pH 7.0) containing 5% milk powder (Bio-Rad).

In order to correlate enamelin expression with amelogenin expression, the blots were differentially probed for both proteins. Briefly, blocked membranes were incubated for 1 h with the anti-enamelin IgGs (see below for full details) diluted 1:1,000 in TBS containing 0.05% Tween (TBST). After washing (3 × 5 min in TBST), the blots were incubated for 1 h in anti-rabbit IgG peroxidase conjugate (1:750 dilution; Sigma Chemicals, Poole, UK), washed again, and developed using metal-enhanced 3, 3’ diaminobenzidine substrate (Sigma Chemicals), resulting in brown/black staining. The blot was then reblocked and incubated for 1 h with rabbit antisera against a peptide corresponding to the 12-amino-acid hydrophilic C-terminal telopeptide (1:2,000 dilution; Eurogentec, Southampton, UK). After washing, the blots were incubated for 1 h with anti-rabbit IgG peroxidase conjugate (1:750 dilution; Sigma Chemicals), washed, and developed using 10 mM sodium acetate buffer containing 0.04% 3-amino-9-ethylcarbazole and 0.015% H_2_O_2_, resulting in red staining.

### Enamelin antibody

Rabbit IgGs were raised against the synthetic peptide EEMFEDYEKPKEKDPPK, which corresponds to a sequence lying in the centre of the putative rat 32-kDa enamelin analogue (Fig. 1). The degree of homology between rat and pig enamelins in this sequence was such that the antibody recognized enamelins from both species. The peptide antibody production and affinity purification were carried out commercially by Eurogentec.

## Results

In order to characterize enamelin proteins at all stages of rat incisor enamel development, samples of enamel protein from each developmental stage were subjected to SDS-PAGE and dual-stain western blotting using anti-32 kDa enamelin and anti-amelogenin telopeptide Igs. Fig. 2 shows an SDS-polyacrylamide gel and a western blot of the total proteins obtained from enamel, representing S1, S2, T, M1, M2, and M3. The SDS-polyacrylamide gel shows the typical range of Coomassie Blue-stainable proteins present in developing rat enamel, with the most abundant proteins migrating with apparent molecular mass values ranging from 5 to 27 kDa. Most of the stained material is amelogenin protein, comprising nascent molecules and their processing products. Matrix protein is readily detectable in the secretory and transition stages (S1, S2, and T) but is progressively removed from the tissue in the maturation stages (M1 to M3). The corresponding western blot shows clear brown/black enamelin cross-reactivity at 50–150 kDa in the secretory-stage samples (S1 and S2) with the most intensely stained bands migrating around 60–70 kDa. The transition-stage enamel (T) contained very little enamelin migrating above 70 kDa, with most staining being visible at around 60 kDa with a smear of putative enamelin degradation-products migrating between the 37- and 50 kDa molecular mass markers. No enamelin cross-reactivity was detected in maturation-stage enamel samples (M1 to M3). None of the developmental stages contained obvious enamelin proteins migrating at 32 kDa (32 kDa is indicated by the dotted line on the blot). The red/brown staining represents the differential staining of amelogenins exhibiting the C-terminal telopeptide. These represent newly secreted amelogenins (as removal of the telopeptide is the first step in the extracellular processing of amelogenin), ranging from approximately 17 to 30 kDa. As with the enamelins, nascent amelogenins began to decline during transition and eventually became undetectable in the maturation-stage tissue (M1 to M3).

**Fig. 3:**
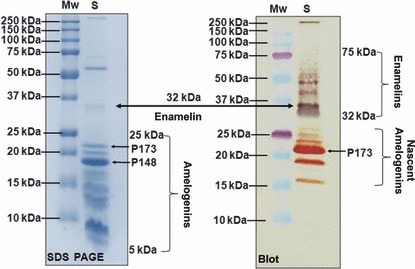
SDS-PAGE and western blotting of porcine secretory-stage enamel matrix proteins. The SDS-polyacrylamide gel shows the characteristic pattern of porcine enamel matrix proteins, including the amelogenins P148 and P173. The western blot is dual stained for both enamelin (brown/black staining) and nascent amelogenin (red staining). The brown/black stain shows the dominant enamelin species migrating at 32 kDa, as expected. Nascent amelogenin expression, as indicated by the red stain, thus confirms the secretory-stage origin of this enamel sample. Mw, molecular-weight-marker ladder; S, porcine secretory stage enamel proteins.

Fig. 3 shows an SDS-polyacrylamide gel and a dual-stained western blot probed using anti-32 kDa enamelin and anti-amelogenin telopeptide IgGs. The SDS-polyacrylamide gel shows the characteristic pattern of porcine developing enamel proteins with amelogenins dominating the protein profile between the 5- and 25-kDa molecular-mass markers (19). The dominant band migrating just below the 20-kDa molecular-mass marker is the major amelogenin processing product corresponding to residues 1–148 of the porcine amelogenin sequence (P148). The 173-amino-acid precursor to P148 (i.e. P173) is the dominant amelogenin splice product and can be seen migrating above P148. The brown/black staining present between 32 and 75 kDa on the corresponding western blot shows the obvious presence of the 32-kDa porcine enamelin along with several higher-molecular-weight enamelin species. The band migrating above 250 kDa on both SDS-PAGE and western blotting is material that failed to enter the resolving gel. The red staining on the blot indicates the presence of nascent amelogenins and shows several alternatively spliced amelogenin species, including the major splice variant, P173, confirming that the enamel sampled was indeed secretory-stage enamel.

## Discussion

The pig model has been central to investigating the form and function of the developing enamel extracellular matrix. The ease with which large amounts of porcine enamel matrix can be obtained has led to many advances in our understanding of amelogenin, ameloblastin, and enamelin biochemistry. Virtually all published work concerning enamelin has been carried out using the pig model, and the role and perceived importance of the 32-kDa enamelin processing product in amelogenesis in general has grown over the last two decades. However, the cleavage sites responsible for generating the 32-kDa enamelin are not well conserved. For example, Fig. 1 shows the relevant sequence data for 11 species (including human). The cleavage sites generating the 32-kDa in pig are absent from the other species shown. A sequence alignment for all known enamelin sequences is presented in Fig. S1. Out of more than 60 species shown, none are homologous to the pig with regard to the presence of the cleavage sites required to generate the N- and C-termini of the 32-kDa enamelin. An alignment of 36 species, published by Al-Hashimi*et al.* as electronic supplementary material (20), shows the same lack of homology between pig and other species with respect to the cleavage sites generating the 32-kDa enamelin. If this precludes the generation of the 32-kDa enamelin in non-porcine species, then the ability to extrapolate, to all species, the importance and role of the specific 32-kDa enamelin fragment seen in the pig, needs to be reassessed.

In order to investigate this further, we examined the developing enamel matrix of rat enamel with respect to enamelin biochemistry. This included an overall survey of the matrix at each developmental stage in search of a rat 32-kDa enamelin analogue. Our survey of rat enamel using western blotting with an antibody designed to recognize the 32-kDa enamelin analogue in rodents, revealed that the dominant immunoreactive species present were migrating with an apparent molecular mass of 60–70 kDa (Fig. 2). This is in complete contrast to the situation in porcine enamel, where the 32-kDa enamelin accumulates, making it the most dominant enamelin-derived component in the porcine enamel matrix (Fig. 3). It is very clear that the predominant rat enamelins have a higher molecular mass than the dominant enamelins found in porcine enamel matrix, which indicates that enamelin proteolysis in the rat is quite different from that occurring in pig. This may be because the cleavage sites responsible for generating the 32-kDa enamelin are not 100% conserved between pig and rat, and this may impair the activity of rat enamel proteases, such as MMP20, against the rat enamelin molecule.

An alternative/additional reason for the paucity of 32-kDa enamelin in rat enamel may be kinetics. Rat enamel goes through the secretory stage in about a week, after which time all matrix protein is removed as the matrix enters the maturation phase. It may be that the proteolytic activity present during the secretory stage simply does not have sufficient time to process the enamelin to the level of the 32-kDa component before the matrix is completely lost. In contrast, because the enamel is much thicker in pigs, it takes much longer to go through the secretory stage and therefore the secretory-stage proteases have longer to act on the enamelin and effectively reduce all secreted enamelin to the level of the 32-kDa component. Whatever the case, it is clear that rat amelogenesis does not require the accumulation of a 32-kDa enamelin analogue. The differential staining of the blot in Fig. 2 shows that amelogenins exhibiting the C-terminal telopeptide (i.e. nascent amelogenins) and enamelins are co-secreted into the enamel and that both classes of protein are subsequently removed during maturation. This gives some ground to hypotheses suggesting functional interactions between enamelins and amelogenin, but in light of the present data the functional importance of the 32-kDa enamelin as a discrete entity is at least debatable in non-porcine species.

To conclude, differences in the sequence of enamelin between pig and other species, such as rat and human, appear to preclude the generation of a 32-kDa enamelin and its subsequent accumulation in the developing enamel. There is even a possibility that the generation of a 32-kDa enamelin during pig amelogenesis is actually irrelevant and the porcine 32-kDa enamelin may be a non-functional bystander during pig amelogenesis. Pig enamel does not mineralize to the same levels as those observed in other species, with porcine enamel only reaching about 60% mineral by volume (21). It is possible that the atypical generation of a 32-kDa enamelin in pigs may even be associated with these reduced mineral levels in mature porcine enamel.

It is clear that some caution should be exercised when assigning functional significance to the 32-kDa enamelin as a discrete entity across species because in non-porcine species, amelogenesis appears to proceed in its absence. The porcine 32-kDa enamelin is post-translationally modified with significant N-linked glycosylation and phosphorylation, and these post-translational modification sites are well conserved (100% homology among pig, mouse, rat, and human), suggesting that the modifications have a functional role (12). In other species, such as rat, no discrete 32-kDa enamelin molecule is produced, so any functionality related to these translational modifications will be associated with enamelin molecules larger than 32 kDa (e.g. the enamelins detected at around 60–70 kDa in this study). We suggest that caution should be exercised when extrapolating experimental findings based on the porcine 32-kDa enamelin to enamelin function in other species (including humans). The larger enamelins in non-porcine species will still carry an analogue of the 32-kDa enamelin domain, but the additional protein sequences present could influence the functionality and behaviour of this domain. In the light of the data presented here, the pig model appears to be less than ideal when trying to understand enamelin function in humans and other species, whether in relation to normal amelogenesis or in cases of enamelin-linked amelogenesis imperfecta.

## Supporting Information

**Fig. S1.** Amino acid sequence alignment for all known enamelin sequences corresponding to the regions centred of the 32 kDa enamelin cleavage sites.
