# Supplementary material for: Is the 32-kDa fragment the functional enamelin unit in all species?
Source: Eur J Oral Sci. 2011 Dec;119(S1):345–50. doi: 10.1111/j.1600-0722.2011.00869.x (PMC3427898; doi:10.1111/j.1600-0722.2011.00869.x)
Supplement: Supplementary file 1 [file eos0119-0345-SD1.pdf]

Supporting information

Is the 32 kDa fragment the functional enamelin unit in all species?

BROOKES SJ, KINGSWELL NJ, BARRON MJ, DIXON MJ, KIRKHAM J

Leeds Dental Institute, University of Leeds, and
Faculty of Life Sciences and School of Dentistry, University of Manchester, UK.

Fig. S1: An amino acid sequence alignment for all known enamelin sequences corresponding to the regions centred of the 32 kDa enamelin cleavage sites. No other species, apart from the pig, exhibits both the N and C terminal cleavage sites generating the pig 32 kDa enamelin.

ALIGNMENT OF N-TERMINAL CLEAVAGE SITE GENERATING THE PIG 32kDa ENAMELIN WITH ENAMELINS FROM OTHER SPECIES.

```
                                                  N-TERMINAL CLEAVAGE SITE
sp|O97939|ENAM_PIG          PQAFPPFGNGLFPYQQPLWHVP----HRIPP-GYGRPPTSNEEGGNPYFGFFGYHGFGGR  211   ENAM_PIG
tr|Q548P8|Q548P8_MOUSE      KSSPST--MMRPENPQYTMTSL----DQKETEQYNEEDPIDPNEDESFPGQSRWGDEEMN  814   Q548P8_MOUSE
tr|D3ZIB0|D3ZIB0_RAT        KRISTPGTMMQPENPQYTMSSL----DQKETEQYNEEDPIDPNEDESFPGQSRWGDDELS  915   D3ZIB0_RAT
tr|Q8IWP4|Q8IWP4_HUMAN      NYAGNPANLRRKPQGPNKHPVG----TTVAPLGPKPGPVVRNEKIQNPKEKPLGPKEQII  448   Q8IWP4_HUMAN
tr|C8C1Q5|C8C1Q5_PHYMC      ERDDSPNTMRQPENPHYPMNTP----NPKKTIPYNEEDPIDPTGDESFPGQSRWGVVESS  465   C8C1Q5_PHYMC
tr|C8C1R1|C8C1R1_MESBI      ERDDSPNTMRQPENPHYPMNTP----DPKKTIPYNEEDPIDPTGDESFPGQSKWGVVQSS  465   C8C1R1_MESBI
tr|B1ACU8|B1ACU8_BALBO      ERDDSPNRMRQPENPHYPMNTT----DPKKTIPYNEEDPIDPTGDESFPGQSRWGVVESS  110   B1ACU8_BALBO
tr|B1ACU9|B1ACU9_BALED      ERDDSPNRMRQPENPHYPMNTT----DPKKTIPYNEEDPIDPTGDESFPGQSRWGVVESS  110   B1ACU9_BALED
tr|B1ACU6|B1ACU6_MEGNO      ERDDSPNRMRQPEXPHYPMNTT----DPKKTIPYNEEDPIDPTGDESFPGQSRWGVVESS  110   B1ACU6_MEGNO
tr|B1ACU7|B1ACU7_BALPH      ERDDSPNRMRQPENPHYPMNTT----DPKKTIPYNEEDPIDPTGDESFPGQSRWGVVESS  110   B1ACU7_BALPH
tr|C8C1Q6|C8C1Q6_DELLE      ERDDSPNTMRQPENPHYPMNTP----DPKKTIPYNEEDPIDPTGDESFPGQSRWSVAESS  465   C8C1Q6_DELLE
tr|B1ACV3|B1ACV3_PSECS      ERDDSPNTMRQPENPHYPINTP----DPKKTIPYNEEDPIEPTGDESFPGQSRWSVAESS  110   B1ACV3_PSECS
tr|C8C1Q7|C8C1Q7_PHOPH      ERDDSPNTMRQPENPHYPMNTP----DPKKTIPYNEEDPIDPTGDESFPGQSRWSVAESS  465   C8C1Q7_PHOPH
tr|C8C1Q8|C8C1Q8_TURTR      ERDDSPNAMRQPENPHYPMNTP----DPKKTIPYNEEDPIEPTGDESFPGQSRWSVAESS  465   C8C1Q8_TURTR
tr|C8C1R0|C8C1R0_PONBL      ERDDSPNTMRQPENPHYPMNTP----DPKKIILYNEEDPIDPTGDESFPGQSRWGVAESS  465   C8C1R0_PONBL
tr|C8C1Q9|C8C1Q9_INIGE      ERDDSPNTMRQPENPHYPMNTP----DPKKTIPYNEEDPIDPTGDESFPGQSRWGMAESS  465   C8C1Q9_INIGE
tr|C8C1R2|C8C1R2_PLAMN      ERDDSPNTMRQPENPHYPMNTP----DPKKTIPYNEEDPRDPTGDESFPGQSRWDVAESS  465   C8C1R2_PLAMN
tr|C8C1R3|C8C1R3_HIPAM      ERDDSPNTMRQPENPHYPMNTP----DPKETVPYNEEDPIDPNGDEPFPGQSRWGVAESS  465   C8C1R3_HIPAM
tr|B1ACV0|B1ACV0_BALMY      ERDVSPNTMRQPENPHYPMNTP----DPKKTIPYNEEDPIDPTGDESFPGQSRWGVAESS  110   B1ACV0_BALMY
tr|B1ACV2|B1ACV2_EUBJA      ERDVSPNTMRQPENPHYPMNTP----DPKKTIPYNEEDPIDPTGDESFPGQSRWGVAESS  110   B1ACV2_EUBJA
tr|B1ACV1|B1ACV1_EUBAS      ERDVSXNTMRQPENPHYPMNTP----DPKKTIPYNEEDPIDPTGDESFPGQSRWGVAESS  110   B1ACV1_EUBAS
tr|B1ACV7|B1ACV7_HEXLI      ERDDSPNTMRQPENPHYPMNTP----DPKETVPYNEEDLIDPNGDEPFPGQSRWGVAESS  110   B1ACV7_HEXLI
tr|B1ACW1|B1ACW1_TAYTA      ERDDSPNTMGRPENPHYPVNIP----DPKETIPYNEEDPIDPTGDEHFPGQSRWDVEELS  110   B1ACW1_TAYTA
tr|B1ACV8|B1ACV8_OVIDA      ERNDPHNTMGQPENPHYPMNTP----DPKETIPYNKKDPNDPTGDEPFPGQTRWGVEKSN  110   B1ACV8_OVIDA
tr|B1ACV9|B1ACV9_ANTAM      ERNDSPNTMGQPENPHYPMNTP----NPKETIPYNKEDPVDPTGDEPFPGQTRWGVEESN  110   B1ACV9_ANTAM
tr|B1ACW0|B1ACW0_TRANA      ERDDSPNTMGQRENPHYPMNTP----DMKERIPYKEEDPVDPTGDEPFPGQSRWGVEKSS  110   B1ACW0_TRANA
tr|C8C1R5|C8C1R5_LAMGL      ERDDSPNTVRQPENPHYPMNTP----DPKETIHYNEEDPTDPTGDEPFPGQSRWGAEESS  461   C8C1R5_LAMGL
tr|B1ACW3|B1ACW3_LAMGU      ERDDSPNTVRQPENPHYPMNTP----DPKETIHYNEEDPTDPTGDEPFPGQSRWGAEESS  110   B1ACW3_LAMGU
tr|B1ACW2|B1ACW2_CAMDR      ERDDSPNTVRQPENPHYPMNTP----DPKETIHYNEEDPTDTTGDEPFPGQSRWGVEESS  110   B1ACW2_CAMDR
tr|C8C1S9|C8C1S9_ERIEU      ERDDSPNTVRQPENPLYPMNTP----DPKDTVPYNEEDPADPTGDETYLGQSQWGMEESN  466   C8C1S9_ERIEU
tr|B2L7U6|B2L7U6_PANTR      NYAGNPANLRRKPQGPNKHPVG----TTVXXXXXXPGPVVRNEKIQNPREKPLGPKEQII  448   B2L7U6_PANTR
tr|B2L7U5|B2L7U5_PANPA      NYAGNPANLRRKPQGPNKXXXX----XXXXXXXXXPGPVVRNEKIQNPREKPLGPKEQII  448   B2L7U5_PANPA
tr|B2L7U8|B2L7U8_9PRIM      NYAGNPANLRRKPQGPNKHPXX----XXXXXXXXXXXXXXXXEKIQNPREKPLGPKEQII  448   B2L7U8_9PRIM
tr|B2L7V2|B2L7V2_NASLA      EEIPSPAKEHFPAGRNTWDHQE----SSPPFKERPGRQEEHLPHPSHGSRGSVFYPEYNP  530   B2L7V2_NASLA
tr|B2L7V0|B2L7V0_HYLSY      EEIPSPAKEHFPAGRNTWDHQE----ISPPFKEDPGRQEEHLPHPSDGSRGSVFYPEYNP  550   B2L7V0_HYLSY
sp|Q9NRM1|ENAM_HUMAN        EEIPSPAKEHFPAGRNTWDHQE----ISPPFKEDPGRQEEHLPHPSHGSRGSVFYPEYNP  605   ENAM_HUMAN
tr|B2L7U0|B2L7U0_PAPAN      ERDDSPNTMGQKESSLYPINTP----DHKETVPYNEEDPIDPTGDEVFPGQNRWGEELSF  498   B2L7U0_PAPAN
tr|B2L7U4|B2L7U4_COLAN      ERDDSPNTMGQKESPLYPINTP----DHKETVPYNEEDPTDPTGDEVFPGQNRWGEELSF  481   B2L7U4_COLAN
tr|B2L7V5|B2L7V5_LAGLA      EREDSPNTMRQKGSPLYPINTP----DQKETVPYNEEDPTDPTGDEYFPGQNRWGEELSF  415   B2L7V5_LAGLA
tr|B2L7V9|B2L7V9_SAGLB      EREDSPNTMGGKESLLYPINTP----DQKETVPYNEEDPIDPTGDEYFPGQYRWGEELSF  414   B2L7V9_SAGLB
tr|C8C1S6|C8C1S6_ELEMA      ERGDSLNTMRQPENPLYPVNTP----NQKETVPYDEEDPIDPTGDEHFPGPGRWAKEESS  467   C8C1S6_ELEMA
tr|C8C1S7|C8C1S7_LOXAF      ERGDSLNTMRQPENPLYPVNTP----NQKETVPYDEEDPIDPTGDEHFPGPGRWAKEESS  467   C8C1S7_LOXAF
tr|C8C1S5|C8C1S5_PROCA      ERDNSPNTIRRPETSLYPMNTP----DQKETVPYNEEDPTDPTGDETFLEPGRWGKEESS  466   C8C1S5_PROCA
tr|C8C1S4|C8C1S4_HETBR      ERDNSPNTIRRPETSLYPMNTP----DQKETVPYNEEDPTDPAGDETFLEPGRWGKEESS  466   C8C1S4_HETBR
tr|C8C1S3|C8C1S3_DUGDU      ERGDSPNTMRQPENPLYPVNTL----DQKETIPYNQEDPNDPTGDEHFPGPGRWGKEESS  467   C8C1S3_DUGDU
tr|C8C1S0|C8C1S0_ECHTE      ERGDPPNTRGQPENRLYRMNTP----DRKEIVPYNEEDPIDPTGDEPFPRPGRWGEEESS  469   C8C1S0_ECHTE
tr|C8C1R8|C8C1R8_AMBHO      ERGDLPNAMGQPENPLYPMSNP----NHKETVPYNEEDPIDPTGDEPFPGPGRWGEEESG  460   C8C1R8_AMBHO
tr|C8C1R9|C8C1R9_CHRAS      ERGDSPNTMGQPENPLYPMNTP----NHKETVPYNEEDPTDPTGDEPFPGPGRWGEEESS  460   C8C1R9_CHRAS
tr|C8C1S1|C8C1S1_ELERU      ERGDSPKTVRPPENPLYPMSTP----SQKERVPYNEEDPVDPNGDELFPGQSRWGEEVPS  467   C8C1S1_ELERU
tr|C8C1S2|C8C1S2_9EUTH      ERGNSPKTIGQPQNPLYPMNTP----DQKETIPYNEEDPIDPNGDELFPGQGRWGEEESS  468   C8C1S2_9EUTH
tr|C8C1S8|C8C1S8_MYOLU      ERDDSPNNAGLPEDPLYPMNTP----DPKETVPYNEEDPIDPTGDEPFPGQSRWGVEESR  468   C8C1S8_MYOLU
tr|B1ACW4|B1ACW4_TAPIN      EKNNPPNSMGQPKNPLYPMNTP----EPKETVLYNEEDPVDPTGDEQFPGQSRWAVEDLS  110   B1ACW4_TAPIN
tr|C8C1R6|C8C1R6_HORSE      ERNYSPNTMGRLKNPLYPVNTP----DVKETVLYNEEDPTDPTGGEAFPGQSRWGVEESS  465   C8C1R6_HORSE
tr|C8C1R7|C8C1R7_FELCA      ERDDSPNTMGQPKNSLYPMNTP----DLKETVPYNEEDPIDATGDETFPGQSRWGMEEPS  467   C8C1R7_FELCA
tr|D2HC94|D2HC94_AILME      ERDDSPNTIGQPKNSLYPINTP----ELKETVPYNEQDPVDATGDESFPGQSRWGMEETS  663   D2HC94_AILME
tr|E2R6T1|E2R6T1_CANFA      ERDDSPNTIGQPRNSLYPINTP----ELKETVPYNEEDPIDPTGDETFPGQNRWGMEEPS  679   E2R6T1_CANFA
tr|B2L7V4|B2L7V4_LAGLA      PQGFPPFGNGLFPYQPPPWQIP----QRVPPPGYGRPPISNEEG----------G----  113   B2L7V4_LAGLA
tr|C8C1Q4|C8C1Q4_VOMUR      ERDDFPGTFRPAGQPGNPSYTLNIPSGQRHPSTYNEEDPIDPAGDEIYQGPKDWGKESNF  469   C8C1Q4_VOMUR
tr|D2KBK9|D2KBK9_PSECU      ERNNFPGTFRPAGQPGNPSHSLNTPSDQRQPSTDNDEDPIDPTGDEFYQGPDAWGKESNF  470   D2KBK9_PSECU
tr|D2KBK2|D2KBK2_PETBR      RDDLPGTFRPAGQSGNPSHSLNTPSGQRQPPSDNEEDPIDPTGDELYQGPDTWGKESNFK  469   D2KBK2_PETBR
tr|D2KBJ4|D2KBJ4_ACRPY      ERDDFPNTFRPAGQPGNPSHPLNTPSGQRQPSTYNEEDPIDPTGDEFYQGPDAWGKESNF  469   D2KBJ4_ACRPY
tr|D2KBL5|D2KBL5_TARRO      EREDFPGTFRAAGQXRKPSHFLNTXAGQRQPSTYNEEDPIDPTGDEXYQGPDAWGKESNF  469   D2KBL5_TARRO
```

Several species have potential cleavage site P-L that might be recognised by the protease responsible for generating the N-terminal of the pig 32 kDa enamelin (highlighted in yellow). However, none of these species have the requisite C-terminal cleavage site responsible for generating the 32 kDa enamelin analogue (see below).

ALIGNMENT OF C-TERMINAL CLEAVAGE SITE GENERATING THE PIG 32kDa ENAMELIN WITH ENAMELINS FROM OTHER SPECIES.

```
                                         C-TERMINAL CLEAVAGE SITE
sp|O97939|ENAM_PIG          ------GQGPNPRSNPT--GQN-GPAVNVSGQGVPRSQSPWGPRQ----TIIH----ENY  105  ENAM_PIG
tr|Q548P8|Q548P8_MOUSE      PIGNTGPGPNAGNNPTVQNGVFPPPKVNVSGQGVPKSQIPWRPSQ----PNIYENYP---  325  Q548P8_MOUSE
tr|D3ZIB0|D3ZIB0_RAT        PVGNTGPGPNAGNNPTVLNGVFPLPKVNVSGQGVPKNQIPWRPSQ----PNIYENYP---  398  D3ZIB0_RAT
tr|Q8IWP4|Q8IWP4_HUMAN      ------RMPGFSSKSEEMMRYNQFNFMNGPHMAHLGPFFGNGLPQ----QFPQ----YQM  91   Q8IWP4_HUMAN
tr|C8C1Q5|C8C1Q5_PHYMC      ------VQGSNTVSNPT--AQN-SPAVNVSGQGVPRSQTPRGPSQ----TNIH----ENY  109  C8C1Q5_PHYMC
tr|C8C1R1|C8C1R1_MESBI      ------VQGSNTVSNPT--AQN-SPAVNVSGQGVLGSQTPRGPSQ----TNIH----ENY  109  C8C1R1_MESBI
tr|C8C1Q6|C8C1Q6_DELLE      ------VQGSNTVSNAT--AQN-SPAVNVSGQGVPRSQTPRGPSQ----TNIH----ENY  109  C8C1Q6_DELLE
tr|C8C1Q7|C8C1Q7_PHOPH      ------VQGSNTVSNTT--AQN-SPAVNVSGQGVPRSQTPRGPSQ----TNIH----ENY  109  C8C1Q7_PHOPH
tr|C8C1Q8|C8C1Q8_TURTR      ------VQGSNTVSNPT--AQN-SPAVNVSGQGVPRSQTPQGPSQ----TNIH----ENY  109  C8C1Q8_TURTR
tr|C8C1R0|C8C1R0_PONBL      ------VQGSNTVSNPT--AQN-SPAVNVSGQGVPRSQTPRGPSQ----TNIH----ENY  109  C8C1R0_PONBL
tr|C8C1Q9|C8C1Q9_INIGE      ------VQGSNTVSNPT--AQN-SPAVNVSGQGVPRSQTPRGPSQ----TNIH----ENY  109  C8C1Q9_INIGE
tr|C8C1R2|C8C1R2_PLAMN      ------VQGSNTVSNPT--AQN-SPAVNVSGQGVPRSQTPRGPSQ----TNIH----ENY  109  C8C1R2_PLAMN
tr|C8C1R3|C8C1R3_HIPAM      ------VQRSNTVSNPT--AQN-SPAVNVSGQGVPRSQIPWGPHQ----TNIH----ENY  109  C8C1R3_HIPAM
tr|C8C1R5|C8C1R5_LAMGL      ------GQGPNTVSNPT--AQN-GPVVNVSGQGVPRSQTPWGPSQ----TNFH----ENY  107  C8C1R5_LAMGL
tr|C8C1S9|C8C1S9_ERIEU      ------GPGSNTVNNPA--VQNPSSTVNISGQGIPRSQIPLGRRQ----PNFH----GNY  110  C8C1S9_ERIEU
tr|B2L7U6|B2L7U6_PANTR      ------RMPGFSSKSEEMMRYNQFTFMNGPHMAHLGPFFGNGLPQ----QFPQ----YQM  91   B2L7U6_PANTR
tr|B2L7U5|B2L7U5_PANPA      ------RMPGFSSKSEEMMRYNQFTFMNGPHMAHLGPFFGNGLPQ----QFPQ----YQM  91   B2L7U5_PANPA
tr|B2L7U8|B2L7U8_9PRIM      ------RMPGFSSKSEEMMRYNQFNFMNGPHMAHLGPFFGNGLPQ----QFPQ----YQM  91   B2L7U8_9PRIM
tr|B2L7V2|B2L7V2_NASLA      ------GYHGFGGRPPYYSEEMFEQDFEKPKEEDPPKAESPGTEP----TANS----TVT  174  B2L7V2_NASLA
tr|B2L7V0|B2L7V0_HYLSY      ------GYHGFGGRPPYYSEEMFEQDFEKPKEEDPPKAESPGTEP----TVNS----TVT  193  B2L7V0_HYLSY
sp|Q9NRM1|ENAM_HUMAN        ------GYHGFGGRPPYYSEEMFEQDFEKPKEEDPPKAESPGTEP----TANS----TVT  249  ENAM_HUMAN
tr|B2L7T5|B2L7T5_MACNE      ------PGPNTGNNPPAQNGIVPLPAVNTSGQGGPGSQIPWRPSQ----PNIR----ENH  163  B2L7T5_MACNE
tr|Q4R358|Q4R358_MACFA      ------PGPNTGNNPPAQNGIVPLPAVNTSGQGGPGSQIPWRPSQ----PNIR----ENH  102  Q4R358_MACFA
tr|B2L7T7|B2L7T7_MACMU      ------PGPNTGNNPPAQNGIVPLPAVNTSGQGGPGSQIPWRPSQ----PNIR----ENH  162  B2L7T7_MACMU
tr|B2L7U0|B2L7U0_PAPAN      ------PGPNTGNNPPAQNGIVPLPAVNTSGQGGPGSQIPWRPSQ----PNIR----ENH  142  B2L7U0_PAPAN
tr|B2L7U4|B2L7U4_COLAN      ------PGPNTGNNPPAQNGIVPLPAVNASGQGGPGSQIPWRPSQ----PNIR----ENH  124  B2L7U4_COLAN
tr|B2L7V5|B2L7V5_LAGLA      ----------------------PVPAVNASGQGGPGSQISWRPSQ----PNIR----VNH  54   B2L7V5_LAGLA
tr|B2L7V9|B2L7V9_SAGLB      ----------------------PVPAVNASGQRGPGSQIPWRPSQ----PNVR----VNH  51   B2L7V9_SAGLB
tr|C8C1S6|C8C1S6_ELEMA      ------QGPNPGNDPTSQNG-ISPPTVNVSNQGVARSQIPWRQSH----PNIH----ENY  111  C8C1S6_ELEMA
tr|C8C1S7|C8C1S7_LOXAF      ------QGPNPGNNPTAQNG-ISPPTVNVSNQGVARSQIPWRQSH----PNIH----ENY  111  C8C1S7_LOXAF
tr|C8C1S5|C8C1S5_PROCA      ------HGPNLGSNPTAQNGVISPSTANVSNQGVPRSQNPWGPSY----PNIH----ENY  112  C8C1S5_PROCA
tr|C8C1S4|C8C1S4_HETBR      ------HGPNLGSNPTAQNGVISPSTANVSNQGVPRSQNPWGPSY----PNIH----ENY  112  C8C1S4_HETBR
tr|C8C1S3|C8C1S3_DUGDU      ------HDLNPGSNRTAQNGVISPSTVNVSNQGASRIQIPWGQIH----PNIY----ENY  112  C8C1S3_DUGDU
tr|C8C1S0|C8C1S0_ECHTE      ------HGP--GANPTAQDGVGSPPAVNASNQGGPGNRTPWGPRQ----PNVY----GNY  108  C8C1S0_ECHTE
tr|C8C1R8|C8C1R8_AMBHO      ------NGQ----NPTAQNGVISPPTVNVSNQGVPGSQIPWGPRQ----PNNH----DNY  108  C8C1R8_AMBHO
tr|C8C1R9|C8C1R9_CHRAS      ------NGP----NPTAQNGVISPPTVNISNQGVPGSQIPWGPRQ----PNYH----DNY  108  C8C1R9_CHRAS
tr|C8C1S1|C8C1S1_ELERU      ------PGPNPGSNP--QHGVISPATVNISNQGVPRTQTPWGPRQ----PNIY----ENY  110  C8C1S1_ELERU
tr|C8C1S2|C8C1S2_9EUTH      ------HSPNPEGNPTAQNGVILPTTVNISNQGVPRNHIPWGPSQ----PHIH----GNY  112  C8C1S2_9EUTH
tr|C8C1S8|C8C1S8_MYOLU      ------HGPNTGGNPTAQNGVNPHPAVNVSGQGPPRSQIPWGSGQ----PNIR----ENY  113  C8C1S8_MYOLU
tr|C8C1R6|C8C1R6_HORSE      ------YGPNTVSNRTVQNGVILPPTVNISGQGIPRRQIPWRPSQ----PKIH----ENY  112  C8C1R6_HORSE
tr|C8C1R7|C8C1R7_FELCA      ------HGPNTVSNPTAQNGVISPPTVNVSGQGVPRTQISWGPSQ----PNIY----ENY  111  C8C1R7_FELCA
tr|D2HC94|D2HC94_AILME      ---------------MAQNGVNSPPTINVSGQGVPRTQITWGPSQ----PNIH----ENY  304  D2HC94_AILME
tr|E2R6T1|E2R6T1_CANFA      ------PGPNTVSNPTAQNGVISPATVNISGQGVPRTQISWGPNQ----PNIH----ENY  323  E2R6T1_CANFA
tr|C8C1Q4|C8C1Q4_VOMUR      -------AQNPGNNQGVHPGVHSTPSVNISIHDVLGSQIPQVPSQ----SNIFE------  110  C8C1Q4_VOMUR
tr|D2KBJ7|D2KBJ7_DISPE      -------AQNPGNNQGVHPGVHSTPSVNISIHDVLGSQIPQVPSQ----SNIFE------  110  D2KBJ7_DISPE
tr|D2KBL0|D2KBL0_9META      -------DQNPGINQGVYPGVNSIPAVNVSGHDIQGNQFPQVPSQ----PHVFG------  110  D2KBL0_9META
tr|D2KBL4|D2KBL4_9META      -------DQNPGINQGVYPGVNSIPAVNVSGHDIQGNQFPQVPSQ----PHVFG------  110  D2KBL4_9META
tr|D2KBL2|D2KBL2_9META      -------DQNPGINQGVYPGVNAIPAVNVSGHDIQGNQFPQVPSQ----PHVFG------  110  D2KBL2_9META
tr|D2KBL1|D2KBL1_9META      -------DQNPGINQGVYPGVNSIPAVNVSGHDIQGNQFPQVPSQ----PHVFG------  110  D2KBL1_9META
tr|D2KBL3|D2KBL3_9META      -------DQNPGINQGVYPGVNSIPAVNVSGHDIQGNQFSQVPSQ----PHVFG------  110  D2KBL3_9META
tr|D2KBK5|D2KBK5_PSEPE      -------DQNPGINQGVYPGVNSVPAVNVSGHDIQGNQFPQVPSQ----PHVFG------  110  D2KBK5_PSEPE
tr|D2KBK0|D2KBK0_9META      -------DQNPGISQGVYPGVNSVPAVNVSGHDIQGNQFPQVPSQ----PNVFE------  110  D2KBK0_9META
tr|D2KBJ9|D2KBJ9_9META      -------DQNPGISQGVYPGVNAVPAVNVSGHDIQGNQFPQVPSQ----PNVFE------  110  D2KBJ9_9META
tr|D2KBK9|D2KBK9_PSECU      -------DQNPGNNQGVHPGVNSIPSVNVSGHDIQGSLFPQVPSQ----PNVFE------  110  D2KBK9_PSECU
tr|D2KBK6|D2KBK6_9META      -------DQNPGNNQGVHPGVNSIPSVNVSGHDIQGSLFPQVPSQ----PNVFE------  110  D2KBK6_9META
tr|D2KBK4|D2KBK4_9META      -------DQNPGNNQGVHPGVNSIPSVNVSGHDIQGSLFPQVPSQ----PNVFE------  110  D2KBK4_9META
tr|D2KBK8|D2KBK8_9META      -------DQNPGNNQGVHPGVNVIPSVNVSGHDIQGSLFPQVXSQ----PNVFE------  110  D2KBK8_9META
tr|D2KBK7|D2KBK7_9META      -------DQNPGNNQGVHPGVNSIPTVNVSGHDIQGSLFPQVPSQ----PNVFE------  110  D2KBK7_9META
tr|D2KBK3|D2KBK3_PETNO      -------DQNPGNNQGVHPGVNSVPAVNVSGHDVQGSQFPQVPSQ----PNVFE------  110  D2KBK3_PETNO
tr|D2KBK2|D2KBK2_PETBR      -------DQNPGNNQGVHPGVNSVPAVNVSGHDVQGSQFPQVPSQ----PNVFE------  110  D2KBK2_PETBR
tr|D2KBK1|D2KBK1_9META      -------DQNPGNNQGVHPGVNSVPAVNVSGHDVQGSQFPQVPSQ----PNVFE------  110  D2KBK1_9META
tr|D2KBJ8|D2KBJ8_GYMLE      -------DQNPGNNQGVHPGVNSVPAVNVSGYDVQGSQLPQVPSQ----PNIFE------  110  D2KBJ8_GYMLE
tr|D2KBJ5|D2KBJ5_DACPA      -------DQNPGNNQGVLPGVNSIPAVNVSGHDVQGSQFPQVPSQ----PNVFE------  110  D2KBJ5_DACPA
tr|D2KBJ6|D2KBJ6_DACTR      -------DQNPGNNQGVHPGVNSIPAVNVSGHDVQGSQFPQVPSQ----PNVFE------  110  D2KBJ6_DACTR
tr|D2KBJ4|D2KBJ4_ACRPY      -------VQNPGNNQGVHPGVNSAPAVNVSGHNVPGSQFPQVPSQ----PNVFE------  110  D2KBJ4_ACRPY
tr|D2KBL5|D2KBL5_TARRO      -------DQNPGNNQGVHPGVNSVPAVNISGYDVQGNQFPQIPSQ----PNVFE------  110  D2KBL5_TARRO
```

Hippopotamus (C8C1R3_HIPAM) contains a potential 32kDa enamelin cleavage site R-S (highlighted in yellow) but lacks the required N-terminal cleavage site required to generate a 32 kDa enamelin analogue.
